# Professionalism Milestones Assessments Used by Emergency Medicine Residency Programs: A Cross-sectional Survey

**DOI:** 10.5811/westjem.2019.11.44456

**Published:** 2019-12-19

**Authors:** Christine R. Stehman, Steven Hochman, Madonna Fernández-Frackelton, Emilio G. Volz, Rui Domingues, Jeffrey N. Love, William Soares

**Affiliations:** * Indiana University School of Medicine, Department of Emergency Medicine, Indianapolis, Indiana; † St. Joseph’s University Medical Center, Department of Emergency Medicine, Paterson, New Jersey; ‡ New York Medical College, Department of Emergency Medicine, Valhalla, New York; § David Geffen School of Medicine at UCLA, Harbor-UCLA Medical Center, Department of Emergency Medicine, Los Angeles, California; ¶ Kendall Regional Medical Center, Department of Emergency Medicine, Broward County, Florida; || Lincoln Medical and Mental Health Center, Department of Emergency Medicine, Bronx, New York; # George Washington University School of Medicine, Department of Emergency Medicine, Washington, District of Columbia; ** University of Massachusetts Medical Center-Baystate Health, Springfield, Massachusetts

## Abstract

**Introduction:**

Professionalism is a vital component of quality patient care. While competency in professionalism is Accreditation Council for Graduate Medical Education (ACGME)-mandated, the methods used to evaluate professionalism are not standardized, calling into question the validity of reported measurements. We aimed to determine the type and frequency of methods used by United States (US) -based emergency medicine (EM) residencies to assess accountability (Acc) and professional values (PV), as well as how often graduating residents achieve competency in these areas.

**Methods:**

We created a cross-sectional survey exploring assessment and perceived competency in Acc and PV, and then modified the survey for content and clarity through feedback from emergency physicians not involved in the study. The final survey was sent to the clinical competency committee (CCC) chair or program director (PD) of the 185 US-based ACGME-accredited EM residencies. We summarized results using descriptive statistics and Fisher’s exact testing.

**Results:**

A total of 121 programs (65.4%) completed the survey. The most frequently used methods of assessment were faculty shift evaluation (89.7%), CCC opinion (86.8%), and faculty summative evaluation (76.4%). Overall, 37% and 42% of residency programs stated that nearly all (greater than 95%) of their graduating residents achieve mastery of Acc and PV non-technical skills, respectively. Only 11.2% of respondents felt their programs were very effective at determining mastery of non-technical skills.

**Conclusion:**

EM residency programs relied heavily on faculty shift evaluations and summative opinions to determine resident competency in professionalism, with feedback from peers, administrators, and other staff less frequently incorporated. Few residency programs felt their current methods of evaluating professionalism were very effective.

## INTRODUCTION

Non-technical skills (NTS) such as communication, teamwork, leadership, and professionalism are vital to providing high-quality patient care.^1^–^2^ NTS deficiencies have been associated with conflict, lawsuits, and loss of medical license, leading to a call for integration of formal NTS assessment into residency training.^3^–^5^ In response, the Accreditation Council for Graduate Medical Education (ACGME) developed core competencies for residents to master during training, of which one-third are NTS including professionalism.^6^ The ACGME further expanded the core competencies with the Next Accreditation System (NAS, or Milestone Project), in which each medical specialty created sub-competencies and milestones (levels within the sub-competencies that showed progressive skill development to guide assessment of trainees).^7^–^8^ These NTS milestones were not meant to be assessment tools themselves; rather they were to “inform the use and development” of such tools.^7^

Of all the NTS competencies, professionalism might be both the most important as well as the most difficult to assess.^9^–^11^ The Council of Emergency Medicine Residency Directors (CORD) found that “assessment and outcome measurement of professionalism are fraught with subjectivity and bias.”^12^ Finding standardized milestone-assessment tools that are emergency medicine (EM) specific and easy to use is difficult, causing residency programs to struggle to integrate competencies into their curricula.^7^,^13^ Given this challenge, various CORD workgroups have proposed a number of ways that model behaviors of professionalism could be assessed, including incorporating non-EM tools; however, no standardized recommendation has been established.^12^,^14^–^15^

Given there are no standardized assessment recommendations evaluating professionalism in residency, we sought to determine the prevalence, variability, and self-perceived effectiveness of the methods that United States (US)-based, ACGME-accredited EM residencies currently use to assess the NTS competency of professionalism, divided in EM into the sub-competencies of accountability (Acc) and professional values (PV) (Supplement).^16^

## METHODS

### Design

This was a cross-sectional survey examining the prevalence of assessment methods used by US-based, ACGME-accredited EM residency programs when evaluating the NTS milestones for Acc and PV from July 31 – September 15, 2017.

### Participants

All US-based EM residency programs that were ACGME-accredited and had graduated at least one residency class by July 1, 2017, were included in the study. We compiled the final participant list, which included 185 programs, by searching the American Medical Association FREIDA database; the residency databases of the American College of Emergency Physicians, the Society for Academic Emergency Medicine, and the American Osteopathic Association; and the websites of the individual residency programs.^17^–^20^ Members of the research group used a combination of contact lists and resources to obtain contact information for each program’s clinical competency committee (CCC) chair or program director (PD). While the goal was to directly send the survey to the CCC chair, in cases where we were unable to identify the CCC chair directly, we sent an email to the PD asking them to either forward the survey request to their CCC chair (preferable) or respond to the survey themselves. The CCC chair and PD were selected to participate in the survey as they are most likely to have comprehensive knowledge of their residencies’ PV and Acc assessments, as well as a global view of performance and self-perceived effectiveness of their individual NTS measurements.

**Educational Research Capsule Summary**

What do we already know about this issue?

*There are no established recommended methods for assessing the difficult to define concept of Professionalism, despite its centrality to high quality medical care*.

What was the research question?

What is the spectrum and self-perceived effectiveness of assessing Professionalism in emergency medicine (EM) residencies?

What was the major finding of the study?

*EM residencies mainly rely on faculty opinion to assess professionalism. Few feel they are very effective in this assessment*.

How does this improve population health?

*Standardizing professionalism assessment methods may help decrease variability and perceived effectiveness of resident assessments allowing improved physician performance*.

### Survey Development and Administration

Drawing from previous work by Sullivan et al., and guided by existing core competency literature, the research group, comprised of six emergency physicians (EP) involved in resident education, used an iterative design and revision process over five working sessions to create a cross-sectional survey (Appendix).^9^ This survey explored assessment practice and resident competency in Acc and PV. The survey was piloted twice and modified for content and clarity based on feedback from approximately 15 EPs not involved in the study. The final survey included a combination of multiple-choice and free-text response questions as well as five demographics questions (Supplementary Material). The final survey was sent via email weblink (https://www.surveymonkey.com) to the CCC chair or PD of each program.^21^ Up to two reminders to complete the survey were sent out at two-week intervals. The survey remained open for six weeks before it was closed for analysis.

### Analysis

We summarized results using descriptive statistics. Methods of NTS resident evaluation were stratified by self-perceived effectiveness. Differences in methods by effectiveness were evaluated with Fisher’s exact testing. We performed all statistical testing using R statistical software (The R Foundation, Vienna, Austria).^22^ This study was approved by the institutional review boards of the research group members’ home institutions.

## RESULTS

### Demographics

Of 185 EM residency programs meeting criteria, 121 (65.4%) completed the survey. Respondents included both three- and four-year programs. The table details the demographics of the respondents compared to the all- EM residency programs surveyed. Because of the anonymity of the survey, it is impossible to say which member of program leadership (CCC chair or PD) provided the responses.

### Tools Used to AssessProfessional Value and Accountability

**Figure 2:**
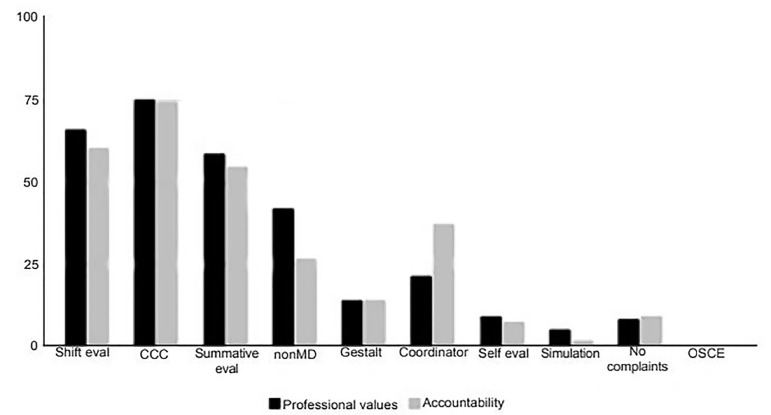
Residency programs’ assessment tools that contribute most to determination of final milestone assessment of professional values and accountability sub-competencies. *eval*, evaluation; *CCC*, clinical competency committee; *OSCE*, Objective Structured Clinical Examination.

The top three assessment tools that respondents indicated are the most important in determining final NTS milestones assessments include CCC opinion (PV 75.2%; Acc 74.4%); faculty shift evaluations (PV 66.1%; Acc 60.3%); and faculty summative evaluations (PV 58.7%; Acc 54.5%). Residency programs used self-evaluations, lack of complaints, simulation, and OSCE less frequently as measurements that contribute to final milestone assessments (Figure 2).

### Self-perceived Effectiveness of Assessments

With regard to self-perceived effectiveness of measurement of NTS milestones, only 11.2% of respondents felt their program was very effective at determining mastery of these sub-competencies, with 48% (54) considering their methods effective, and 40% (49) indicating their evaluation methods are only somewhat effective. For measurement of PV, self-perceived very effective programs more often used feedback from the program coordinator or office staff (85% vs 51%, p = 0.04) as well as non-physician feedback (100% vs 72%, p = 0.04). For measurement of Acc, self-perceived very effective programs also more often used feedback from the program coordinator or office staff (100% vs 62%, p<0.01) as well as simulation (54% vs 24%, p = 0.04). No other significant differences emerged in methods used to assess professionalism in programs that perceived their assessment to be very effective compared to others.

## DISCUSSION

Well-developed NTS, in particular professionalism, are essential to a physician’s ability to deliver effective, compassionate patient care.^2^ Thus, NTS comprise one-third of the ACGME competencies that residents must master in order to graduate. Based on ACGME guidance, each medical specialty divides the core competencies into their own sub-competencies and milestones. Like the creation of specialty-specific milestones, the ACGME offers only guidelines on skill assessment, leaving the methods and tools to the discretion of each residency program.^7^

This study represents the first attempt since the implementation of the core competencies and milestones to quantify the variability and breadth of methods and tools used by US-based EM residencies to evaluate professionalism.

While EM residencies overall appear to incorporate a variety of tools to assess residents in professionalism, faculty opinion, through both on-shift and summative evaluations, contributes most frequently to a resident’s assessment and final milestone placement. These findings are in contrast to how EM PDs have previously assessed residents with potential professionalism issues, which has historically included both emergency department and off-service evaluations, advisor/residency leadership evaluations, and 360-degree evaluations.^9^ Our finding that overall professionalism milestone assessments more frequently favor faculty opinion raises concerns. First, professionalism evaluation benefits from direct observation of behaviors, which faculty do less often as residents advance in training.^23^ Second, non-physician staff and patients may observe different aspects of professionalism than faculty physicians.^24^–^25^ For example, a resident may behave differently in the presence of a supervisor than with a colleague or a patient.^26^

Additionally, our study found that many respondents do not consider their residency programs very effective at assessing professionalism milestones. This finding echoes the results of the 2010 PD survey, which showed that 50.7% of PDs felt their current methods of assessment of professionalism were inadequate.^9^ Although we cannot use perceived self-effectiveness as evidence of objective effectiveness of methods, it is concerning that the faculty charged with evaluating residents for readiness to progress to independent practice do not feel they have “very effective” methods of evaluating professionalism.

Unfortunately, the observed variability, the reliance on faculty opinions, and the limits in self-perceived effectiveness in assessing EM residents’ professional values are likely related to the lack of standardized definitions and evidence-based measurement tools. Adams et al. argued that EM in particular needs to demonstrate commitment to professionalism given the unusual vulnerability of the typical EM patient and the fact that the EP “performs an essential service in a unique social context, possesses specialized skill, and requires the confidence of patients.”^27^ Lack of professionalism in both medical school and residency has been associated with professionalism issues later in a physician’s career.^28^–^30^ Unfortunately, definitions of professionalism vary.^31^ Some state that it cannot be easily and clearly defined while others note that unprofessional behaviors are like the Supreme Court definition of obscene (“I know it when I see it”).^32^–^33^ In EM, Adams et al. does not define professionalism but rather identifies eight fundamental elements of it: (1) suspension of self-interest; (2) honesty; (3) technical competence; (4) authority and accountability; (5) communication; (6) justice; (7) humility; and (8) avoiding misuse of power.^27^

Few validated tools exist to guide assessment of these competencies, leading faculty to rely heavily on gestalt.^34^ This is especially an issue with assessment of professionalism as the definition remains unclear, potentially making assessment a moving target based on which faculty member is evaluating the resident and in what circumstances.^15^,^35^–^36^ CORD, like the ACGME, has suggested including multiple methods to measure professionalism such as using ethics knowledge and moral reasoning tests, multisource feedback (MSF; 360-degree evaluation), direct observation assessment tools, ratings- and survey-based assessment tools (including patient satisfaction surveys), portfolios and narratives, critical incident reporting systems, and simulation.^12^, ^14^–^15^ CORD has also suggested exploring the use of tools developed outside of EM for this purpose.^14^ Despite these recommendations, a recent systematic review of such tools found that the one with the best psychometric properties has not yet been evaluated in either the US or in EM.^37^ LaMantia et al. recently developed a MSF tool that seems to have excellent internal consistency; however, its implementation was quite challenging and time intensive.^38^

Given these limitations in the tools available, it is not surprising that this study demonstrates that some residencies simply provide faculty with the milestones and ask them to rate the residents. A quote from a respondent sums up the problem with this approach:

“The milestones are very broad and nonspecific in their descriptions. Most faculty have NO training in how to properly select a number for a milestone. There is tremendous variance between physicians who grade a single resident.”

This variance will likely exist no matter which tool a residency chooses, especially if there is limited faculty development associated with implementation of the tool. These forms are completed by individuals who essentially become the assessment “tool,” making faculty and staff development imperative to providing quality feedback to residents and residency programs alike.^7^,^39^ Without training on easy to use, validated tools, assessment often goes back to what the assessor knows and does regularly.^34^

Future research should focus on the impact of different assessment tools on predicting future professional assessment. Further, residency programs may benefit from standardized, evidence-based recommendations on the factors that should be included when measuring professional values in resident physicians.

## LIMITATIONS

This study potentially has several limitations. First, the study was not designed to determine the objective “best” or most-effective methods of assessing professionalism. As detailed above, issues with defining and measuring outcomes related to professionalism make objective, validated, specialty-specific assessments rare. That said, even with a lack of evidence-based methods, core faculty are still required to assess a resident’s professionalism and in judging readiness for independent practice. Therefore, our study serves to determine the current landscape and variability in assessment measures, as well as the perceived effectiveness of faculty who are required to use those measures.

Additionally, to avoid duplication only one person at each program was surveyed, and their view of the program may be different than others within their program. However, by choosing the CCC chair or PD, we attempted to select the respondent with the highest likelihood of having experience in ranking residents, up-to-date information on current practice in resident evaluation, and knowledge of current and recently graduated residents. Further, by keeping surveys anonymous, we attempted to promote honest program self-assessments. Second, based on the respondents’ demographic, the respondents provided a diverse representative sample of all EM programs despite not having achieved a 100% response rate. Finally, this study only looked at EM residency assessment of NTS, so the results may not be fully applicable to other specialties. However, it is likely that the results highlight difficulties in assessing professionalism that are present in all medical specialties.

## CONCLUSION

Although a variety of assessments are used overall by EM residencies to evaluate milestones for PV and Acc, the most frequently used measures rely on faculty shift evaluations and summative opinions that, based on prior literature, may only provide a limited assessment of professionalism. Methods that incorporate non-faculty opinions, standardization through simulation or OSCE environments and self-reflection are used less frequently. Further, few residency programs felt their current methods of professional milestone assessment are very effective. Further research and guidelines that assist EM residency programs in standardizing assessments of professionalism incorporating the evidence-based literature that is available may help to decrease residency variability and increase perceived effectiveness.

## Supplementary Information



## Figures and Tables

**Figure 1:**
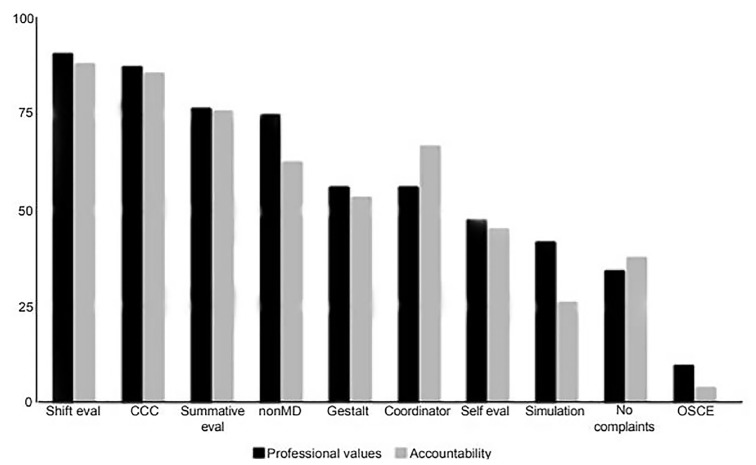
Methods used by residency programs to determine milestone assessment of professionalism sub-competencies, professional values and accountability. *eval*, evaluation; *CCC*, clinical competency committee; *OSCE*, Objective Structured Clinical Examination.

**Table:** Demographics of the respondents’ residency programs.

	Respondents (#)	%	Invited (#)	%
Residency program				
3 year	80	66.1%	132	71.4%
4 year	32	26.4%	51	27.6%
Other	2	1.7%	2	1.1%
No answer	7	5.8%		
Residency program established				
Less than 5 years	19	15.7%		
5–15 years	23	19.0%		
More than 15 years	76	62.8%		
No answer	7	5.8%		
Number of residents per year				
Less than 8	18	14.9%	31	16.8%
8–15	79	65.3%	126	68.1%
Greater than 15	15	12.4%	28	15.1%
No answer	9	7.4%		
Type of hospital				
Community	34	28.1%		
University	62	51.2%		
County	9	7.4%		
Other	16	13.2%		
Geographic location				
Northeast (CT, MA, ME, NH, NY, RI, VT)	27	22.3%	35	18.9%
Central East (IN, KY, MI, OH, TN)	20	16.5%	34	18.4%
Mid-Atlantic (DC, DE, MD, NC, NJ, PA, VA, WV)	20	16.5%	40	21.6%
North Central (AR, IA, IL, KS, MN, MO, ND, NE, OK, SD, WI)	14	11.6%	24	13.0%
Southeast (Puerto Rico, AL, FL, GA, LA, MS, SC)	11	9.1%	17	9.2%
Southwest (AZ, CO, NM, NV, TX, UT)	13	10.7%	18	9.7%
West (CA, ID, MT, OR, WA, WY)	11	9.1%	18	9.7%
No answer	5	4.1%		
Percent of graduates achieving Accountability level 4 milestones				
Greater than 95%	44	36.4%		
75% – 95%	59	48.8%		
50% – 75%	8	6.6%		
Less than 50%	7	5.8%		
Percent of graduates to achieve Professional Values level 4 milestones				
Greater than 95%	49	40.5%		
75% – 95%	59	48.8%		
50% – 75%	6	5.0%		
Less than 50%	4	3.3%		
