# Supplementary material for: Professionalism Milestones Assessments Used by Emergency Medicine Residency Programs: A Cross-sectional Survey
Source: West J Emerg Med. 2019 Dec 19;21(1):152–9. doi: 10.5811/westjem.2019.11.44456 (PMC6948707; doi:10.5811/westjem.2019.11.44456)
Supplement: Supplementary file 1 [file wjem-21-152-s001.docx]

| **Appendix 1: Survey tool on methods of assessment for Professionalism Sub-competencies** |
| --- |
| 1. Please indicate all the tools your program uses to evaluate the Professional Values milestone for a resident.   - Self-Evaluation - Faculty Shift Evaluation - Faculty Summative Evaluation (monthly or less frequent) - Non MD Clinical Input (e.g. RN, NP, PA, Clerk, Tech, Patient) - Program Coordinator/Office Staff - OSCE - Simulation Scenarios - Clinical Competency Committee Opinion - Gestalt - Lack of Complaints - Other (please specify) |
| 2. Please indicate all the tools your program uses to evaluate the Accountability milestone for a resident.   - Self-Evaluation - Faculty Shift Evaluation - Faculty Summative Evaluation (monthly or less frequent) - Non MD Clinical Input (e.g. RN, NP, PA, Clerk, Tech, Patient) - Program Coordinator/Office Staff - OSCE - Simulation Scenarios - Clinical Competency Committee Opinion - Gestalt - Lack of Complaints - Other (please specify) |
| 3. What are the TOP 3 tools that you believe contribute to the resident's final evaluation for Professional Values?   - Self-Evaluation - Faculty Shift Evaluation - Faculty Summative Evaluation (monthly or less frequent) - Non MD Clinical Input (e.g. RN, NP, PA, Clerk, Tech, Patient) - Program Coordinator/Office Staff - OSCE - Simulation Scenarios - Clinical Competency Committee Opinion - Gestalt - Lack of Complaints - Other (please specify) |
| 4. What are the TOP 3 tools that you believe contribute to the resident's final evaluation for Accountability?   - Self-Evaluation - Faculty Shift Evaluation - Faculty Summative Evaluation (monthly or less frequent) - Non MD Clinical Input (e.g. RN, NP, PA, Clerk, Tech, Patient) - Program Coordinator/Office Staff - OSCE - Simulation Scenarios - Clinical Competency Committee Opinion - Gestalt - Lack of Complaints - Other (please specify) |
| 5. Do you believe the usefulness of these tools varies by training year?   - Yes - No |
| 6. Does your program use a rubric to determine if each resident meets the appropriate milestone?   - Yes - No |
| 6a. If yes, please describe the rubric your program uses to assess non-technical skills                  milestones. |
| 7. How effective is your program in determining whether residents meet the milestones for non-technical skills?   - Not at all effective - Somewhat effective - Effective - Very effective |
| 8. What percentage graduating residents meet ALL level 4 criteria for Accountability?   - < 50% - 51 - 75% - 76-95% - > 95%              The level 4 milestones for Accountability are:   - Can form a plan to address impairment in one’s self or a colleague in a professional or confidential manner - Manages medical errors according to principles of responsibility and accountability in accordance with institution policy |
| 9. What percentage graduating residents meet ALL level 4 criteria for Professional Values?   - < 50% - 51 - 75% - 76-95% - > 95%              The level 4 milestones for Professional Values are:   - Develops and applies a consistent approach to evaluating appropriate care, possible barriers and strategies to intervene that consistently prioritizes the patient's best interest in all relationships and situations. - Effectively analyzes and manages ethical issues in compacted and challenging clinical situations |
| 10. If you are comfortable doing so, please upload the evaluation tools your program uses. |
| 11. Please describe the way in which you perceive the variance in usefulness of evaluation tools. |
| 12. How many years of training do your residents complete?   - 3 years - 4 years - Other |
| 13. What type of hospital is your residency program based in?   - University Hospital - Community Hospital - County Hospital - Other |
| 14. How many residents are recruited each year?   - Less than 8 - Between 8-15 - More than 15 |
| 15. Where are you located?   - Northeast (CT, MA, ME, NH, NY, RI, VT) - Mid Atlantic (DC, DE, MD, NC, NJ, PA, VA, WV) - Southeast (Puerto Rico, AL, FL, GA, LA, MS, SC) - Central East (IN, KY, MI, OH, TN) - North Central (AR, IA, IL, KS, MN, MO, ND, NE, OK, SD, WI) - Southwest (AZ, CO, MN, NV, TX, UT) - West Coast (CA, ID, MT, OR, WA, WY) |
| 16. How long has your residency program been accredited by ACGME?   - Less than 5 years - Between 6-15 years - More than 15 years |
